# Primary Healthcare in Sudan: Challenges, Shortfalls, and How the Healthcare Workforce Is Navigating the Lack of Resources and Healthcare Planning

**DOI:** 10.1002/puh2.70160

**Published:** 2025-10-29

**Authors:** Mohammed SaifAldeen Osama Hassan Abdelrahamn

**Affiliations:** ^1^ Faculty of Medicine University of Khartoum Khartoum Sudan

**Keywords:** conflict, healthcare accessibility, healthcare planning, healthcare workforce, primary healthcare

## Abstract

With the ongoing Sudanese conflict, the healthcare system deteriorated immensely. Primary healthcare particularly faces numerous challenges of adaptation, with issues such as influx of displaced people, limited supplies and funds, haphazardous planning, and absent infrastructure affecting the accessibility, operations, and quality of care afforded for the communities they serve. Despite these conditions, healthcare professionals remain committed and are able to maneuver through very challenging conditions in order to provide care. Swift interventions are imperative to alleviate the situation in Sudan to prevent collapse of the healthcare system, starting with the cease of conflict and investing in primary healthcare processes to further enable and support the efforts of healthcare professionals.

## 1 Manuscript

Prior to the beginning of the ongoing armed conflict in Sudan, the country's healthcare system faced several obstacles in building and sustaining a functioning framework of operation. This can be observed in the abundance of policies, strategies, and plans that were unmatched by the required implementation and channeling of necessary funding [1]. These weaknesses were further exacerbated by the breakout of the war, which severely disrupted healthcare planning and decision‐making and drastically strained healthcare funding [2].

Primary healthcare centers are considered the first point of contact with the healthcare system for the majority of the Sudanese population, with it sometimes being the only accessible healthcare service provision level for many [3]. Despite this, even before the current war, primary healthcare in Sudan was underpinned by many challenges, including inaccessibility, improper governance and planning, substandard quality, and insufficient investment [4]. These conditions were intensely deteriorated by the conflict breaking out in April of 2023 [5]. According to the WHO, in the Sudanese capital of Khartoum, only 16% of primary care centers are operational [6], leading to a drastic reduction in essential healthcare services access across conflict‐affected states.

Through this commentary, we aim to highlight the day‐to‐day challenges faced by the healthcare system. This was done through a visit to a primary healthcare center in one of the rural neighborhoods in Shendi, a city that is near the conflict‐stricken capital Khartoum. The healthcare workforces of the center were interviewed, and the working conditions of the center were observed.

One of the noted major determinants of the healthcare provision in the center was the number of people that it provides services for. Since the beginning of the conflict, conflict‐free areas have received an overwhelming number of internally displaced people (approximately 7.1 million since April 2023) [7]. This has led to the straining of the already scarce health resources in those areas, as well as an increased burden of disease due to the overcrowding of individuals, substandard living conditions, and inaccessibility of essential services [8].

When interviewing the medical director of the center, they noted that there is a scarcity of the healthcare workforce, with only three individuals—a medical doctor, a lab technician, and a midwife—holding an official appointment by the ministry. Several healthcare professionals do sometimes help in service provision in a volunteering capacity; however, that doesn't last for a long period due to lack of an official appointment status. The deficiency in the healthcare workforce is a result of the insufficient funding to cover their cost [9, 10], as well as Sudanese healthcare professionals migrating and seeking employment abroad due to the attacks and violence against healthcare professionals and unfavorable working conditions [5]. Those who do choose to stay—in cases such as this center—incentivized by their desire to serve their own communities, find themselves handling multiple job descriptions in order to provide a comprehensive healthcare experience. An example of such a case is the medical director of the center, who undertook the responsibility of the position while being the only general practitioner in the center. In addition, due to the absence of a pharmacist, the medical director is also responsible for dispensing medication, as well as the procurement and purchasing processes of pharmaceutical products. Tasks like this carry extra burdens given the context of the war, with components of the health system, such as medications and pharmaceuticals, facing more challenges due to the overwhelming demand and supply shortages [11].

Another issue highlighted was the infrastructure deficiencies. For example, due to the unstable electrical supply, the lab technician is forced to sometimes work entire shifts performing tests using a phone's flashlight as a light source for the light microscope. On the other hand, the volunteer vaccination technician recounted once having to refrigerate vaccines at home overnight due to the power outage at the center. The impact of the inconsistent power supply cannot be understated, and the issues that result from it are faced on even larger scales in conflict‐affected states [12].

From the patients’ side, over the span of 4 days, 35 people were surveyed about the quality of care they received at the center and their satisfaction with it. The individuals surveyed were all the patients who came to receive care throughout those 4 days and gave their verbal consent to be surveyed. The survey results revealed that the community residents depend on it for a range of essential services. The results also showed that although the center mainly serves locals from the neighborhood, several individuals from other neighborhoods seek care in the center due to certain services being unavailable in more proximal health facilities and the internally displaced population within the area. Such results pose new challenges in that primary healthcare by definition orients its services to the context and needs of its population [13], and with the influx of internally displaced people and deterioration of healthcare accessibility, it becomes a struggle to navigate new diseases and outbreaks within the community and catering to a diverse and large number of patients [13, 14].

When asked about the satisfaction level with the quality of the services provided, 60% and 37.1% reported a score of 5 and 4, respectively, on a (1–5) scale, indicating favorable reviews of the patients’ experiences. A majority highlighted the competence and supportive attitude of the healthcare professionals at the center, despite the limited service provided. This underscores the dedication of the healthcare workforce to finesse these conditions and provide for the community, despite the varying challenges to healthcare provision in both conflict‐affected and conflict‐free areas in Sudan [15]. This makes healthcare workers one of the few standing pillars that are preventing the Sudanese healthcare system from collapsing.

## 2 Conclusion

In conclusion, urgent action is needed to support healthcare provision in Sudan, starting with immediate plans to cease the conflict, focusing on replenishing primary healthcare facilities, and capitalizing on competent healthcare professionals. This needs to be spearheaded by developing sensitive and timely plans and strategies, properly allocating both internal funds and external monetary support, as well as developing resilience mechanisms and frameworks that can better sustain future impact.

## Author Contributions

Mohammed SaifAldeen Osama Hassan Abdelrahamn worked on conception, design of the work, original drafting and writing, and reviewing of the commentary.

## Funding

The author has nothing to report.

## Ethics Statement

The commentary was developed from data and site visits carried out as part of the approved community medicine curriculum of the University of Khartoum—Faculty of Medicine. Approval on the site visit and collected data was obtained from the community medicine department.

## Consent

In addition, informed consent was obtained from each participating person prior to interviews or conduction of surveys.

## Conflicts of Interest

The author declares no conflicts of interest.

## Data Availability

The datasets used and/or analyzed during the current study are available from the corresponding author on reasonable request.
